# Suspension of Criminal Proceedings for Perpetrators of Intimate Partner Violence Against Women: Impact on Re-Entries

**DOI:** 10.3389/fpsyg.2021.725081

**Published:** 2021-10-29

**Authors:** Paulo Vieira-Pinto, José Ignacio Muñoz-Barús, Tiago Taveira-Gomes, Maria João Vidal-Alves, Teresa Magalhães

**Affiliations:** ^1^Department of Forensic Sciences, Pathology, Gynaecology and Obstetrics, Paediatrics, Institute of Forensic Sciences, University of Santiago de Compostela, Santiago de Compostela, Spain; ^2^IINFACTS - Institute of Research and Advanced Training in Health Sciences, Department of Sciences, CESPU, University Institute of Health Sciences (IUCS), Gandra, Portugal; ^3^Faculty of Medicine, University of Porto, Porto, Portugal; ^4^CINTESIS - Centre of Health Technology and Service Research, University of Porto, Porto, Portugal; ^5^Faculty of Health Sciences, Fernando Pessoa University, Porto, Portugal

**Keywords:** reduce offender recidivism, intimate partner violence, integrated consensual solution, criminal justice system, provisional suspension of criminal proceedings

## Abstract

Intimate partner violence (IPV) is one of the most prevalent crimes in our society, but the legal mechanisms to oppose it are recent. The Portuguese Provisional Suspension of Criminal Proceedings (PSCP) as a criminal justice system (CJS) response proposes an integrated consensual solution with the involved parties, to reduce offenders’ recidivism. This article analyses the effect of PSCP on re-entries into the CJS. We examined 1,662 IPV police reports, exploring cases that underwent PSCP and re-entries of the same offender in the CJS. Results show that PSCP is applied in 17% of the cases. From all analyzed determinants, with a possible relation to the PSCP implementation, it was found that social violence and the age of both victims and defendants emerge as significantly associated with the request or acceptance of this legal mechanism. No variables tested moderated the relationship between PSCP and re-entry over 96months following the first police report. The article also examined variables that might moderate the decision to request this legal mechanism among victims and defendants.

## Introduction

### Intimate Partner Violence

Intimate partner violence (IPV) is one of the most common health threats in our society. It is characterized by violent actions (physical, emotional, psychological or sexual, among others) against a person, perpetrated by his/her former or current partner, regardless of cohabitation (WHO, 2012), and occurs in all cultures and countries (WHO, 2000; Garcia-Moreno et al., 2006; Ellsberg et al., 2015).

The consequences of IPV affect negatively personal health outcomes, but also the familial and socioeconomic aftermaths (WHO, 2013). IPV affects specially women and, even though male victims of IPV exist, and face somewhat similar challenges, and IPV has had long been a gendered-based problem (WHO, 2013).

A report of the WHO states that 15 to 71% of all women suffer from physical and/or sexual abuse from an intimate partner at least once during their lifetime (Garcia-Moreno et al., 2006). In the European countries, the reality it’s not different. In the European Union Agency for Fundamental Rights’, the Violence Against Women 2014 survey disclosed that one in three women in Europe has experienced at least one act of physical, psychological, or sexual violence since the age of 15 (FRA, 2014).

Portugal also presents a concerning portrait of this phenomenon. In 2019, a total of 29.498 IPV reports were made to the Portuguese criminal justice system (CJS), (RASI, 2019) exposing a high yearly rate (2.7/100.000 inhabitants). Since IPV perpetrated by male partners against female victims is the most frequent and complex scenario found in Portugal, it seems important to examine whether legal measures aimed at this phenomenon are successful (Vieira-Pinto et al., 2021).

In Portugal, since 2001, IPV is considered a public crime. This means that, upon acknowledgement of its occurrence, the Public Prosecution Service, regardless of who reported it, files criminal proceedings whether or not the victim agrees. Police and government employees are mandatory reporters whenever they become aware of IPV in the line of their duties or because of such duties (article 242, Portuguese Penal Procedure Code). Additionally, any citizen can report it to the judicial authority.

Nonetheless, it has been advocated that IPV victims must not be “merely passive receptors” of the CJS response (Cramer, 2004) and are to be included in the violence deterrence efforts, namely, by being accounted for during the decision-making process. This promotes their well-being (Wemmers, 2008; Finn, 2013) and safety perception. This may also promote victim’s empowerment and, therefore, their recovery from trauma (Herman, 2015). Besides, it ensures that not only justice is served, but also social censorship against IPV is roused, which concurs to prevent further events (Cramer, 2004; Römkens, 2006; Bagwell-Gray et al., 2015). Bearing this in mind, how can the CJS promote the victim’s safety, reduce revictimization, and take the victim’s will into account in the judicial process?

### The Current Study

Finding effective methods of reducing the problem has proven extremely difficult. Given the importance of the use of legal measures with potential to reduce revictimization, this study aimed at exploring the following questions: What is the implementation rate of the PSCP mechanism? Are there differences between cases that undergo PSCP from those that do not (e.g., demographics, type of violence, and risk factors)? What impact does this implementation have in deterring new IPV-related re-entries into the CJS?

### Revictimization, Re-Entries in CJS and Recidivism

When a victim is repeatedly exposed to violent behavior by an intimate partner, this is conceptualized as revictimization, having as reference the subject of violence: the victim (Tolman and Wang, 2005; Goodman and Epstein, 2008). However, this revictimization is not always acknowledged by the CJS, since not all cases of revictimization are reported.

If an IPV victimization is reported to the CJS, then this is considered an entry, and consequently, all following entries of the same victim-offender dyad (under a report of IPV to the CJS) will be considered a re-entry. This is so, regardless of what the previous court decision may have been.

In contrast, recidivism, according to the Portuguese Penal Code (article 75), happens when the accused, after being convicted to a prison sentence of 6months or more, is sentenced again to a similar verdict for a new crime committed in less than 5years. The number of re-entries is always less than the number of revictimization cases because the victim is often unwilling or unable to report the abuses to the authorities. Nonetheless, considering that the CJS works solely with reported crimes, the number of re-entries, although underrepresenting the phenomenon, offers a close depiction of the revictimization cases.

### Strategies to Deter IPV

Substantial policy improvements, addressing IPV eradication, have been developed all over the world at the end of the last century (Klein, 2004; Kotanen, 2017; Prenzler and Fardell, 2017; Chester and DeWall, 2018).

The most frequent responses for this problem include victim support services, such as emergency shelters and psychological support. Victims demand justice and expect legal actions against the offender to increase their protection from future harm (Garner and Maxwell, 2009), so they rely on CJS to trigger proper mechanisms.

In the nineties, mandatory prosecution policies regarding IPV treated it as a serious crime [Ferraro (Ferraro and Pope, 1993; Hanna, 1996)] and focused both on mandatory prosecution measures (e.g., mandatory arrest and no-drop prosecution) and promoting proactive responses (Goodman and Epstein, 2008; Nichols, 2014). This has been upheld as a useful deterrent of IPV *via* punishing the offender and by promoting the victim’s feeling of safety (Klein and Crowe, 2008). Within these legal responses, the IPV defendant can face prosecution or undergo a community-based program such as pretrial diversion programs.

Pretrial diversion programs, as a community coordinated response strategy, may be a good alternative to mandatory prosecution, since the offenders are placed under a supervision program (Ulrich, 2002). Involving offenders in the development and implementation of solutions that focus on their own personal and social rehabilitation tends to promote a positive behavior change (Tutty et al., 2020) and, thus, promote victims’ protection (King and Batagol, 2010).

Community coordination response strategy programs are a concept used by professionals who intervene in IPV. Its main objective is to integrate a continuous collaboration response within the organizations and services and to provide a sustained and organized response between the agencies of a community (Pennington-Zoellner, 2009).

One of the first community responses to IPV that was implemented came in Duluth, Minnesota, in the 1980s. The main objectives of community coordination in cases of IPV are safety for survivors and responsibility for perpetrators (Mills et al., 2013).

Currently, community coordination response to IPV recommend interventions, including policies that encourage/determine the arrest and prosecution of offenders, its referral to an intervention program (BIP), and their monitoring by parole officers (Shepard and Pence, 1999).

It is noteworthy, for comparison purposes, that research has shown that the involvement of offenders in their rehabilitation, for example, *via* BIP, has a more positive impact on violence reduction due to self-volition toward behavior change (Tutty et al., 2020).

In Europe, a percentage of self-referred of 22% was found (Hamilton et al., 2013) and in Portugal, 62.5% of non-court-mandated offenders in BIP (Cunha and Gonçalves, 2019). This program persuades defendants to complete the program rather than facing prosecution, to reduce recidivism. Indeed, several researchers have established that court-mandated involvement of the offender in pretrial diversion programs reduces the high dropout rate and promotes positive changes both in attitudes and in behavior (Mills et al., 2013; Tutty and Babins-Wagner, 2019; Arce et al., 2020; Tutty et al., 2020).

This also has been asserted by the Council of Europe (2009) who emphasized the absolute need to specifically address offenders in IPV cases. Thus, Portugal and neighboring countries, such as Spain and France, have included diversion solutions into their criminal justice law so to provide appropriate interventions to offenders requiring treatment or other services.

In Spain, the approach called *La Conformidad* (article 801, Ley de Enjuiciamiento Criminal) allows faster justice operation, provided that the offender agrees with the prosecution. He/she may see his/her sentence reduced by a third or replaced by a suspension of its execution. In these cases, the offenders may be subjected to supervised intervention after conviction (Nogales, 2014).

In France, the legal system also allows the choice of offender referral to follow-up programs (Oddone, 2020). Through a set of protective measures, French Law allows the implementation of a judicial scrutiny imposition, called *Contrôle Judiciaire*, by submitting the aggressor to compulsory psychosocial surveillance (article 138.17, French Code of Criminal Procedure).

However, measures to re-socialize the offender have been proven difficult to put into practice (Polsby, 1992; Bennett et al., 1999; Bell et al., 2013). Simultaneously, most CJS policies to deter IPV are in non-observance of the victims will (Cramer, 2004; Goodman and Epstein, 2008; Bell et al., 2013).

### Provisional Suspension of Criminal Proceedings

In Portugal, since 1987, there is a legal mechanism, named Provisional Suspension of Criminal Proceedings (PSCP), intended to ensure an active role of victims in the criminal investigation and decisions. It also allows an integrated consensual solution between the involved parties, targeting the defendant’s resocialization and the prevention of revictimization. It usually applies to criminal misdemeanors. In these cases, initiative departs from the Public Prosecutor, differently from IPV.

In IPV cases, according to article 281 of the Penal Procedure Code, PSCP implementation can be ordered by the Public Prosecution Service when all the following conditions are fulfilled: (a) free and clear request of the victim to PSCP implementation; (b) agreement from the defendant; (c) agreement from the Investigating Judge; (d) crime not aggravated by the result (e.g., death or serious physical injury); and (e) inexistence of previous conviction of the defendant or previous PSCP implementation for similar crimes (toward the same or another victim). Upon PSCP implementation, the defendant must abide by a set of duties or rules of conduct (e.g., compensate the victim; abstain from residing in defined places or regions; attend previously determined programs or activities; and refrain from specific behaviors, depending on the case) for a maximum period of 5years. If the defendant complies, the case is dismissed.

In cases of non-implementation of this measure, the defendant faces prosecution and trial, yet, in Portugal, IPV is punishable with a prison sentence of 5years but the conviction rate, according to national data, is low (10%; RASI, 2019).

### PSCP Determinants

The PSCP implementation request by the IPV victim must be made freely and clearly but all involved parties must agree. It is important to acknowledge whether risk factors identified in the literature were considered in this decision (Campbell et al., 2008; Messing et al., 2017; Herbert et al., 2021). Risk factors may include the type of violence inflicted, firearm access, personal perception of risk by victims, jealousy and possessiveness, stalking and threats, mental disorder, substance abuse, and previous criminal record (Rodrigues et al., 2021; Vieira-Pinto et al., 2021). In the present study, only those available in criminal records were considered.

PSCP focuses on the defendant in the presentential phase. It offers an opportunity for behavior change and social reintegration and is seen as an additional tool to reduce recidivism. It is thus relevant to identify IPV risk factors that were identified in the defendants.

PSCP is a legal mechanism and was planned to promote consensus in interventions and ensure the protection of the victims’ legal assets during criminal proceedings. It also aims to promote the defendant’s resocialization, as defined in article 40 of the Portuguese Penal Code and reduce the legal procedures’ duration.

It also aims to guarantee conflict resolution between victim and defendant and avoid a revictimization process.

Once the Portuguese CJS takes notice that someone is a victim of IPV, criminal proceedings are automatically triggered. The victim is dragged into a process that she/he does not control. The prosecution does not stop despite the victim’s will, although the victim participates as a witness and interested party (Goodman and Epstein, 2008; Bell et al., 2013). This gives the victim a relevant role, namely, in the process of considering alternative solutions to promote the defendant’s rehabilitation and prevent recidivism, instead of being put on trial.

There is no other published research carried out in Portugal that focuses on the PSCP or on its ability to deter and rehabilitate IPV defendants. However, according to the official data, the PSCP applicability rate in DV cases increased over the last 3years: In 2017, the PSCP was employed in 1.998 DV cases, then, in 2018, to 2.486 and, in 2019, to 2.630 DV cases (RMP, 2019).

PSCP is a legal mechanism used by the Portuguese CJS that aims to deter criminal activity, including IPV crime. This legal mechanism may be implemented for a maximum period of 60months. Since this implementation does not occur in the exact moment of the crime commitment and only afterward, during the criminal proceedings, our research team extended the follow-up period to 96months so that it would identify as much re-entries as possible. The goal of this study was to respond: Is the Portuguese PSCP able to contribute to reducing re-entries into the CJS in IPV cases?

Specifically, we aim to answer three interrelated research questions: (a) which is the PSCP implementation rate; (b) may we compare characteristics, types of violence inflicted by defendants, namely physical, psychological, sexual, social or economic violence, and defendant risk factors, such as substance abuse, and weapons possession of IPV-related cases with a PSCP implementation request, as opposed to other forms of CJS intervention; (c) once applied, will the PSCP reduce IPV re-entries into the CJS in a 96-month follow-up period?

## Materials and Methods

### Study Design and Scenario

The present study is a retrospective cohort analysis. The sample comprises male defendants who allegedly committed IPV crimes against women, whether as current or former intimate partners.

**Figure 1:**
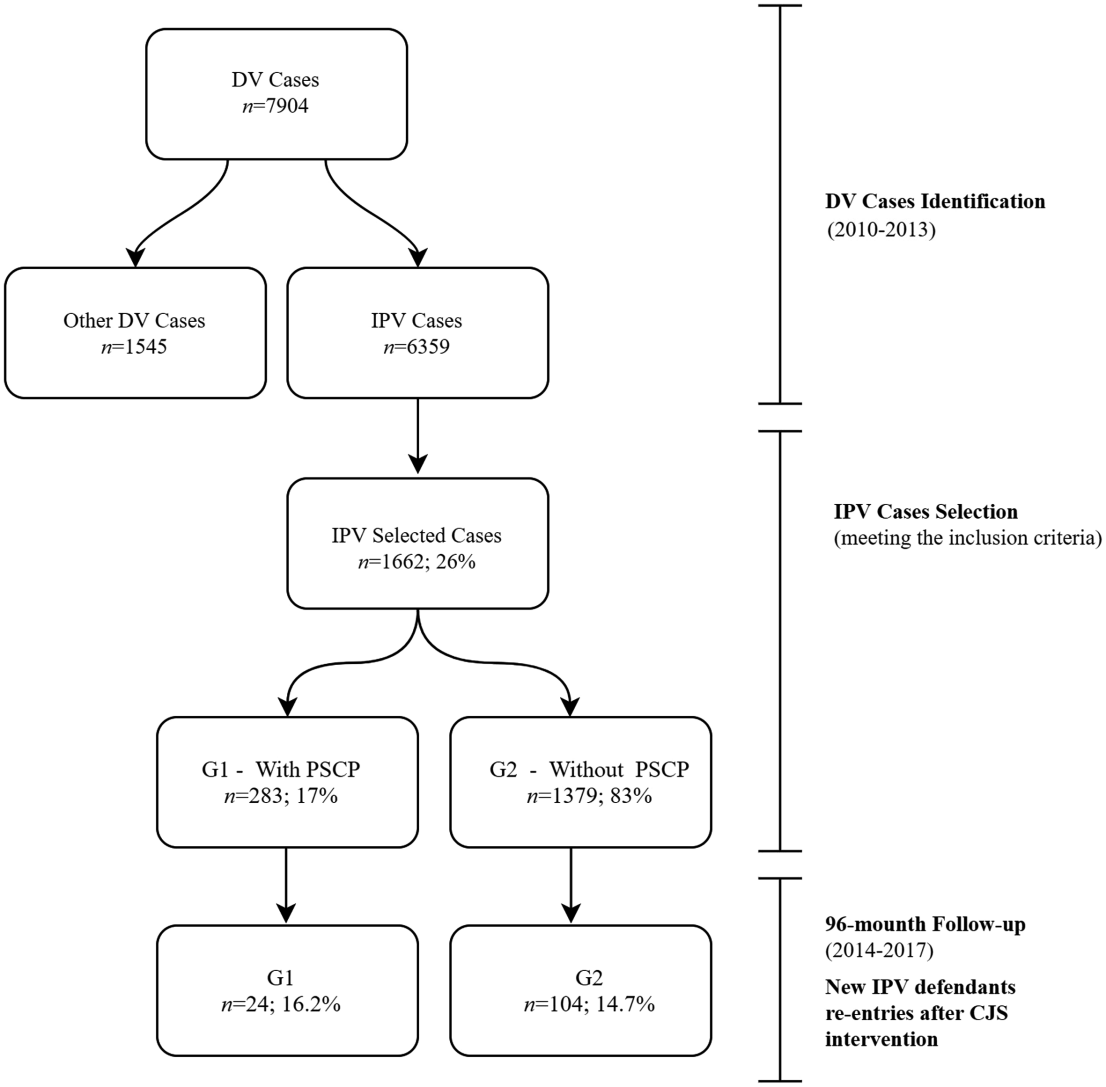
Study design.

The source of information for the present study was the IPV database of the *Guarda Nacional Republicana* (GNR), one of the Portuguese Security Forces. IPV cases were selected concerning the period between January 1, 2010, and December 31, 2013 (*n*=1,662). These cases were subsequently cross analyzed with the Public Prosecution Service’s IPV database, to check whether the selected cases had undergone PSCP. Two groups were then created, considering this last feature: G1 – With PSCP (*n*=283); G2 – Without PSCP (*n*=1,379) – Figure 1. In a second phase, the GNR IPV database was scanned for cases that occurred from January 1, 2014, to December 31, 2017, and re-entries by earlier identified defendants were screened for. A 96-month follow-up was provided (Figure 1).

### Participants

For the present study, the inclusion criteria were the following: (a) cases of alleged IPV (between intimate partners, whether former or current, in dating relationships, married couples, or similar, with or without cohabitation); (b) complaint(s) presented by the victim, always regarding the same offender, at GNR police stations in Porto; (c) victim: female, aged 16 or older; (d) alleged offender: male, aged 16 or older, since, in Portugal, criminal liability applies only to citizens who are 16 older; (e) fully available identification of victims and defendants to allow CJS re-entries tracking within the same region. All IPV cases occurred in Porto district, in the North of Portugal, which holds one of the highest IPV rates in the country (RASI, 2019).

Several studies have been conducted to identify factors that are considered IPV predictors [Bouchard (Campbell, 2002; Sani et al., 2020; Bouchard and Wong, 2021; Rodrigues et al., 2021)]. Normally, studies list two broad categories of variables: (a) demographic variables and (b) violence-related factors (Daly and Pelowski, 2000; Taft et al., 2004; Chambers et al., 2008).

Research has consistently shown that factors, such as younger age, lower socioeconomic status, unemployment, education, being unmarried, substance abuse, violence in one’s family of origin, history of relationship conflict, and prior severity of assaults, are considered as predictors of recidivism risk behavior [Bell (Campbell, 2002; Campbell et al., 2007; Bell et al., 2013; Messing et al., 2017)]. Therefore, several demographic variables were included to determine the extent to which the effects of age, education, employment, and marital status, among victims and offenders, may influence their decision process to request PSCP legal mechanism.

Violence-related variables were also considered in the present study. Frequently, researchers have assessed that risk factors such as alcohol or drug use, weapon possession, type and severity of violence inflicted and children’s exposure to DV have been acknowledged as a risk factor of IPV recidivism (Daly and Pelowski, 2000; Campbell et al., 2007, 2008; Chambers et al., 2008; Bullock et al., 2021).

The victim’s and the defendant acceptance of the PSCP implementation was used as an independent variable, which served to divide the groups. As a dependent variable, we use the first re-entry into the CJS after that legal mechanism was enforced.

### Data Analysis

Statistical analysis was conducted using the R programming language (Team R C, 2014). The study sample was divided into G1 (With PSCP) and G2 (Without PSCP) groups, and its characteristics were described using categorical variables. The pattern of missing data was assessed using Little’s missing completely at random (MCAR) test using LittleMCAR package (Little, 1988). Data imputation was performed using the Multivariate Imputation by Chained Equations (MICE) package (Van Buuren, 2018). Variables were imputed using a proportional odds model. All reported variables were considered in the imputation model. Data imputation was repeated 100 times. To assess the robustness of the results, the same analysis was performed with the removal of incomplete cases relevant to each test. The Chi-square test was used to assess differences in distributions between categorical variables. Predictors for PSCP were modelled using multivariate logistic regression on imputed data. All reported variables were used as covariates in the model. For this purpose, all categorical variables were converted into dummy binary variables. The effect of PSCP in recidivism was assessed using a crude cox regression model. The model considered as independent observations of all timespans between episodes of reporting. Cases in which PSCP was implemented started follow-up at that time. Significance was considered at *p*<0.05.

This study was reviewed and approved by the Health Ethics Committee of the *Centro Hospitalar de S. João/Faculdade de Medicina da Universidade do Porto*.

## Results

### Population Characterization

The age distribution of female victims and male defendants, as well as their professional status, is described in Table 1. Considering age, the average in both groups, with and without PSCP application (G1 and G2, respectively), was as follows: (a) victims – G1=43.3years (18–86; *SD*=12.06), G2=44years (18–89; *SD*=12.17), *p*=0.029; (b) defendants – G1=46.25years (19–86; *SD*=12.38), G2=46.08years (18–92; *SD*=12.16), *p*=0.035.

**Table 1:** Victims’ and defendants’ demographics.

		Victim’s characteristics					Defendant’s characteristics				
		With PSCP G1(*n*=283)		Without PSCP G2(*n*=1,379)		*p* ^*^	With PSCP G1(*n*=283)		Without PSCP G2(*n*=1,379)		*p* ^*^
		*n*	%	*n*	%		*n*	%	*n*	%	
Age (years)	≥ 16–20	8	2.8	10	0.7	0.029 (0.029)	2	0.7	3	0.2	0.035 (0.035)
	21–30	37	13.1	166	12.0		35	12.4	127	9.2	
	31–40	70	24.7	382	27.7		51	18.0	321	23.3	
	41–50	95	33.6	418	30.3		82	29.0	438	31.8	
	51–60	50	17.7	246	17.8		69	24.4	287	20.8	
	61–64	11	3.9	43	3.1		16	5.7	47	3.4	
	≥ 65	10	3.5	82	6.0		16	5.7	105	7.6	
	Missing	2	0.7	32	2.3		12	4.2	51	3.7	
Employment status	Employed	95	33.6	365	26.5	0.250 (0.250)	104	36.8	402	29.2	0.439 (0.439)
	Unemployed	99	35.0	463	33.6		102	36.0	450	32.6	
	Missing	89	31.5	551	40.0		77	27.2	527	38.2	

p* values presented for both imputed (former) and raw model (latter).

There was a larger rate of victims who were in a current relationship both in groups with PSCP application and without it [G1=91.9% (*n*=260); G2=88.1% (*n*=1,224); *p*=0.124]. As such, married and in-union victims had higher PSCP rate application in both groups: (a) married - G1=70.3% (*n*=199), G2=62.7% (*n*=864); (b) In-union – G1=20.5% (*n*=58), G2=25.1% (*n*=346); (c) Dating – G1=1.1% (*n*=3), G2=1.0% (*n*=14). There were no significant differences between groups. The defendant’s risk factors, such as alcohol abuse and weapon possession, revealed differences between groups with PSCP application and without (*p*=0.003 and *p*<0.001, respectively) – Table 2. Relevant differences were found between groups, regarding economic and social violence (*p*=0.002 and *p*<0.001, respectively) – Table 3.

**Table 2:** Defendants risk factors.

		With PSCP G1 (*n*=283)		Without PSCP G2 (*n*=1,379)		*p* ^*^
		*n*	%	*n*	%	
Alcohol abuse	Yes	145	51.2	529	38.4	0.003 (0.003)
	No	94	33.2	532	38.6	
	Missing	44	15.6	318	23.1	
Drug abuse	Yes	18	7.4	64	4.6	0.432 (0.432)
	No	212	74.9	976	70.8	
	Missing	53	18.7	339	24.6	
Weapon possession	Yes	201	71.0	814	59.0	<0.001 (<0.001)
	No	82	29.0	565	41.0	
	Missing	0	0.0	0	0.0	

p* values presented for both imputed (former) and raw model.

**Table 3:** Types of violence registered and children’s exposure to DV.

		With PSCP G1 (*n*=283)		Without PSCP G2 (*n*=1,379)		*p* ^*^
		*n*	%	*n*	%	
Physical	Yes	200	70.7	899	65.2	0.225 (0.225)
	No	63	22.3	347	25.2	
	Unknown	20	7.1	133	9.6	
Psychological/emotional	Yes	202	71.4	901	65.3	0.157 (0.157)
	No	61	21.6	345	25.0	
	Unknown	20	7.1	133	9.6	
Sexual	Yes	8	2.8	22	1.6	0.262 (0.262)
	No	253	89.4	1,223	88.7	
	Unknown	22	7.8	134	9.7	
Economic	Yes	29	10.3	74	5.4	0.002 (0.003)
	No	225	79.5	1,171	84.9	
	Unknown	29	10.3	134	9.7	
Social violence	Yes	29	10.3	59	4.3	<0.001 (<0.001)
	No	227	80.2	1,186	86.0	
	Unknown	27	9.5	134	9.7	
Children’s exposure to DV	Yes	141	49.8	636	46.1	0.149 (0.146)
	No	105	37.1	587	42.6	
	Missing	37	13.1	156	11.3	

p* values presented for both imputed (former) and raw model (latter).

### PSCP Determinants

The factors perceived as a determinant for PSCP implementation are described in Table 4. Of all analyzed variables, three presented statistically significant correlations.

**Table 4:** PSCP determinants.

			OR	[95% CI]	*p*
Age	Victim		0.95	[0.90–1.00]	0.036
	Defendant		1.06	[1.01–1.12]	0.029
Relationship with the alleged offender	Marital	Current	0.48	[0.13–21.79]	0.277
		Past	0.35	[0.06–2.03]	0.239
	In-union	Current	0.27	[0.07–1.09]	0.066
	Dating	Current	0.69	[0.05–10.23]	0.789
		Past	1.74	[0.07–43.98]	0.735
Unemployment status	Victim		1.34	[0.79–2.28]	0.284
	Defendant		0.86	[0.51–1.46]	0.576
Types of violence registered	Physical		1.52	[0.82–2.80]	0.183
	Psychological/emotional		1.7	[0.88–3.27]	0.112
	Sexual		0.81	[0.12–5.54]	0.833
	Economic		1.37	[0.55–3.41]	0.494
	Social		1.5	[1.01–6.63]	0.047
Defendants risk factors	Alcohol abuse		1.48	[0.88–2.49]	0.143
	Drug abuse		1.87	[0.67–5.25]	0.234
	Weapon possession		1.32	[0.20–8.73]	0.776
	Cold weapon use		0.51	[0.16–1.59]	0.245
Severity of injuries presented in victims	Need for hospitalization Unknown		0	[0.00–0.00]	0.985
	Without violence-related injuries		0.89	[0.52–1.55]	0.693
Other tested variables	Children’s exposure to IPV		1.03	[0.59–0.59]	0.909
	Assault in public spaces		0.73	[0.25–2.15]	0.573

OR – Odds Ratio; CI – confidence interval.

As depicted in the current results, the younger the victim, the more likely the case to undergo PSCP [*OR*=0.95; *CI*=(0.9, 1.0); *p*=0.036]. Besides, the defendant’s age and the odds of PSCP implementation are directly proportional [*OR*=1.06; *CI*=(1.01, 1.12); *p*=0.029]. On the other hand, social violence [*OR*=1.5; *CI*=(1.01, 6.63); *p*=0.047] was the only significant statistical association to PSCP request.

Data presented in Table 5 illustrate the effect of PSCP implementation on re-entries over time. The PSCP implementation group (G1) had a mean follow-up time of 288days (*SD*=281.705), and in 148 cases, 24 turned out to be re-entries. In the non-PSCP implementation group G2, the mean follow-up period was of 2,838days (*SD*=1200.819). Of the 767 defendants involved in this study, 104 had re-entered the CJS.

**Table 5:** CoxPH regression model for Survival effect on re-entries in CJS.

Variable	Subcategory		G1 - With PSCP			G1 - Without PSCP		
			HR	[95% CI]	*p*	HR	[95% CI]	*p*
Age	Victim		0.98	[0.94–1.01]	0.140	1.02	[0.95–1.09]	0.600
	Defendant		1.01	[0.98–1.04]	0.507	1.00	[0.94–1.07]	0.940
Relationship with the alleged offender	Marital	Current	1.14	[0.49–2.64]	0.768	0.28	[0.07–1.06]	0.060
		Past	0.76	[0.21–2.79]	0.680	0.00	[0.00–0.00]	<0.001
	In-union	Current	0.27	[0.07–1.09]	0.066	0.53	[0.18–1.60]	0.260
	Dating	Current	1.20	[0.51–2.82]	0.670	0.00	[0.00–0.00]	<0.001
		Past	0.00	[0.00–0.00]	<0.001	0.00	[0.00–0.00]	<0.001
Unemployment status	Victim		1.02	[0.66–1.55]	0.945	1.08	[0.44–2.69]	0.860
	Defendant		0.80	[0.51–1.25]	0.326	0.30	[0.13–0.69]	<0.001
Defendants risk actors	Alcohol abuse		1.69	[1.08–2.64]	0.022	2.64	[0.83–8.36]	0.100
	Drug abuse		1.30	[0.68–2.47]	0.431	1.56	[0.24–10.13]	0.640
	Weapon possession		1.78	[0.98–3.25]	0.059	1.55	[0.45–3.37]	0.490

No significant association was found between PSCP and the decrease of IPV re-entries, except for alcohol abuse by the defendant in G1 (*p*=0.022) and defendant unemployment status in G2 (*p*=<0.001). As such, defendants, who received a PSCP application and had a history of alcohol abuse, presented a higher risk of re-entry. On the other hand, unemployed defendants tend to have a lower acceptance rate of PSCP.

## Discussion

In this study, three main objectives were outlined: Firstly, it was tested whether individual factors related to social-demographic variables of women and their partners were significantly associated with the PSCP implementation rate. Secondly, an analysis was made on whether several defendant individual factors, such as type of violence inflicted (physical, psychological, sexual, social, or economic) and risk factors (substance abuse, possession of weapons), are weighted in the PSCP implementation request; Thirdly, once applied, it was tested whether PSCP reduces IPV re-entries into the CJS in the 96-month follow-up period.

Information from two databases was used, which allowed us to perform this third analysis during the studied period.

### PSCP Implementation Rate

The present study shows that PSCP was used only in 17% of the cases of IPV. Several explanations may be advanced to such a low rate: (a) the victim’s unawareness about this legal mechanism, and, according to the Law, the PSCP implementation only occurs at the victim’s request; (b) the defendant’s refusal to accept PSCP and its mandatory conditions, which is one of the legal requirements; (c) the defendant previous benefit of PSCP, for another DV crime; (d) the Public Prosecution Service’s decision to prosecute the defendant come what may, given the nature and severity of the case, for anticipating their failure if subjected to pretrail rehabilitation measures.

Hester and Lilley (2014) consider that programs focusing on the defendants’ risk factors, such as their chemical dependency, and DV programs, are vital for an integrated and comprehensive approach to prevent and combat IPV. These authors defend that such strategies should be part of a national strategy or policy. Access to effective interventions that tackle IPV offenders must be available, not to take the role of the existing criminal justice, but to combine efforts to address persistent patterns of male violence in intimate relationships.

In Portugal, despite the current results, PSCP could be an opportunity to relieve the pressure on the prosecution, trial and even prison, by promoting rehabilitation and by re-socializing the defendant. It also may promote active participation from victims who so wish.

### Determinants With a Possible Relation to the PSCP Implementation

It is very difficult to determine which variables play a key role in the decision-making process of all involved parties (victim and defendant) concerning the request and acceptance of PSCP. From all analyzed determinants with a possible relation to the PSCP implementation, three variables emerge in the present study as significantly associated with this legal mechanism: (a) the presence of social violence (*p*=0.047); (b) victim’s age (*p*=0.036); (c) offender’s age (*p*=0.029).

Social violence can be characterized as a set of attitudes and behaviors of the offender that aim to control the victim’s life. These forms of violence have increasingly raised the attention of the scientific community, namely, the concept of coercive control, characterized by the offender’s control over the victim using constant intimidation and instilling permanent fear (Stark, 2007; Barlow et al., 2020). This was found to be more prevalent in heterosexual relationships, where men impose and manipulate women. Our study showed that victims of social violence are more likely to achieve PSCP (*p*=0.047), which may be the result of the victim’s perception of the severity of the crime, among others. Victims who suffer physical or psychological aggression may seek responses, such as prosecution and trial of the defendant, other than solutions such as rehabilitation programs, as is the case of PSCP.

There appears to be an important association between the age of the female victim and her decision-making process. It was found that the younger the victims, the more likely the application of PSCP (*p*=0.029). Probably, younger victims may request this application because they believe in the defendant’s rehabilitation and ability to change if assisted by the PSCP mechanism. Older victims, on the other hand, seem less prone to believe in the defendant’s rehabilitation, possibly for having lived long-lasting abusive relationships (Wilke and Vinton, 2005). As shown in other studies, older victims may prefer different solutions, such as leaving the abusive relationship or going directly to trial (Shurman and Rodriguez, 2006; Alexander et al., 2009).

Among defendants, it was observed that the PSCP was applied more often to older offenders (*p*=0.035). This has also been observed in studies about this subject (Karakurt et al., 2019; Lila et al., 2019). This outcome suggests that the aging of the offenders may be a personal characteristic that leads them to accept batterer intervention programs as an alternative to other legal measures, such as facing trial. Older offenders may also develop greater awareness and responsibility for their violent acts as well as be more open to welcome alternative strategies to violence in interpersonal relations. Conversely, the younger offenders may hold a challenging attitude toward authority figures, which makes them less receptive to understand and accept consensus solutions such as PSCP.

It would be expected to find further variables associated with defendant risk factors, such as alcohol consumption or weapons possession. It is known that substance abuse triggers aggressive behavior, adding the risk of new relapses, for example, excessive alcohol consumption increases by eight times the risk of physical/psychological abuse and twice the risk of intimate partner attempted or consummated murder (Campbell et al., 2008; Foran and O'Leary, 2008; Moore et al., 2008; Cunha and Gonçalves, 2019; Spencer and Stith, 2020). Weapon possession is also considered an important risk factor (Messing et al., 2017). However, our results suggest that the involved parties (CJS, victim, and defendant) may not perceive these variables as a determinant for PSCP. Moreover, the Public Prosecution Service may consider that stricter measures may be more effective in specific cases due to the risk assessed.

The present study also reveals a concerning number of children exposed to IPV (more than 45%). This makes IPV cases an even more severe situation (Carter et al., 2020), requiring stouter measures from the CJS to effectively stop violence and protect victims.

### PSCP Effect on Re-Entries in CJS

Results show that PSCP implementation does not seem to contribute to deterring new re-entries into the CJS within the 96months following the first police report (Table 5). Of the 283 defendants subject to PSCP implementation, 16.2% re-entered the CJS (Figure 1). This may sustain the hypothesis that not all defendants who are suspects of committing IPV are similar (Holtzworth-Munroe and Meehan, 2004; Johnson, 2008), and CJS and social services interventions targeting offenders inadequately privilege the use of a one-size-fits-all approach (Gross et al., 2000; Cramer, 2004; Goodman and Epstein, 2008). This finding is supported by the results of other studies (Flinck and Paavilainen, 2008; Lila et al., 2014; Cunha and Gonçalves, 2019), which consider that some IPV offenders tend to minimize or fail to recognize acts of violence for which they have been prosecuted. Therefore, the implementation of different legal measures should be considered by the CJS for these cases so that PSCP has an effective rehabilitating effect instead of representing guilt exoneration.

It must be noted that the re-entry figures point that there are defendants who may be related to more than one case, in both groups. These results are consistent with other research about the escalation of severity of harm inflicted on victims (Bland and Ariel, 2015; Barnham et al., 2017) which reinforced the identification of a phenomenon described as “The power few” (Sherman, 2007). This refers to a small number of offenders being possibly responsible for a large proportion of IPV crimes reported to the CJS. This study disclosed a re-entry rate reduction after PSCP implementation.

It is important to note that this study does not address IPV against male victims or female offenders nor does it include homosexual dyads for practical reasons. This was decided considering the aim of testing legal responses to a sample that represents the most frequently found dyads in the studied population, based on the fact that gender inequities (male dominance over women) have been the substance of maintaining IPV for ages (Reed et al., 2010). Even though this study aims to lay the foundation for an effective approach to IPV against women by male offenders, future research must broaden to other aspects of IPV, including mutual violence, female offenders, same-sex relations, or others.

Although the choice of demographics as moderator was based on the intention to identify variables easily recognized as influencing victims’ decision to request PSCP, this limited our ability to assess the underlying mechanisms of our conclusions.

Future research should also study potential moderators and mediators which allow the judicial system to understand whether the victims ask for PSCP measure voluntarily or under the influence of the defendant. It also should be cleared whether defendants accept this just to avoid trial or to be rehabilitated.

Besides, although our analytical approach took into account the initial differences between groups (e.g., the severity of violence), other qualitative differences, such as the perpetrator’s motivation to be violent, among others, should be considered.

Ultimately, the current results should be read with proper caution since they are based on the log of records of GNR of Porto and any defendant that may have re-entered the CJS in other regions is not here accounted for, yet such cases are expected to be rare. Besides, the generalizability of the results should be assessed in further analysis with data encompassing other regions. Also, re-entries that run off the period of the current study were not accounted for. Also, not all IPV risk factors were considered, since limited to those present in criminal records. Yet, these records focus on the more severe and more often related to violent behavior and impulsiveness as is the case of drug and alcohol abuse. Future studies might consider analyzing the decision-making processes and heuristics used by Public Prosecution Service to refer an IPV case for mandatory supervision.

Results revealed that from all determinants that could be related to PSCP implementation, the presence of social violence, victim’s, and the defendant’s age was identified as significantly associated with it. We did not find, however, evidence to demonstrate that the application of the PSCP could contribute to a decrease in re-entry into the CJS.

Observing the re-entry cases, it was found that 24 defendants re-entered the CJS after the application of PSCP laying the foundation for future research on whether such individuals should have undergone PSCP and the whys of such decision-making by the Public Prosecution Service.

This study did not identify any characteristics either in victims who requested PSCP as well as in those who did not. Both groups show more similarities than differences. The same happened within the defendants. Considering the trust that is placed in the CJS response, it is important to continue to identify the cases and contexts in which CJS interventions might be likely to deter future abuse.

## Data Availability Statement

The data analyzed in this study is subject to the following licenses/restrictions: Data not available due to legal restrictions. Requests to access these datasets should be directed to porto.pgd@tribunais.org.pt.

## Ethics Statement

This study was reviewed and approved by the Health Ethics Committee of the Centro Hospitalar de S. João/Faculdade de Medicina da Universidade do Porto. Written informed consent from the patients/participants OR patients/participants legal guardian/next of kin was not required to participate in this study in accordance with the national legislation and the institutional requirements.

## Author Contributions

PV-P conceived the presented idea and organized the database. JM-B and TM contributed to the conception and design of the study. TT-G performed the statistical analysis. PV-P wrote the manuscript in consultation with MV-A, who wrote sections of the manuscript. JM-B helped to supervise the project. TM supervised and coordinated the study and the findings of this work. All authors contributed to the article and approved the submitted version.

## Funding

Partially supported by Competitive Reference Groups Help ED431C 2017/05. Council of Education and Culture, Xunta de Galicia, Galicia, Spain.

## Conflict of Interest

The authors declare that the research was conducted in the absence of any commercial or financial relationships that could be construed as a potential conflict of interest.

## Publisher’s Note

All claims expressed in this article are solely those of the authors and do not necessarily represent those of their affiliated organizations, or those of the publisher, the editors and the reviewers. Any product that may be evaluated in this article, or claim that may be made by its manufacturer, is not guaranteed or endorsed by the publisher.
